# Leveraging a Phage-Encoded Noncanonical Amino Acid: A Novel Pathway to Potent and Selective Epigenetic Reader Protein Inhibitors

**DOI:** 10.1021/acscentsci.3c01419

**Published:** 2024-02-28

**Authors:** Peng-Hsun Chase Chen, Xuejiao Shirley Guo, Hanyuan Eric Zhang, Gopal K. Dubey, Zhi Zachary Geng, Carol A. Fierke, Shiqing Xu, J. Trae Hampton, Wenshe Ray Liu

**Affiliations:** † Texas A&M Drug Discovery Center and Department of Chemistry, Texas A&M University, College Station, Texas 77843, United States; ● Department of Biochemistry, Brandeis University, Waltham, Massachusetts 02453, United States; § Department of Pharmaceutical Sciences, Texas A&M University, College Station, Texas 77843, United States; ∥ Institute of Biosciences and Technology and Department of Translational Medical Sciences, College of Medicine, Texas A&M University, Houston, Texas 77030, United States; ⊥ Department of Biochemistry and Biophysics, Texas A&M University, College Station, Texas 77843, United States; # Department of Cell Biology and Genetics, College of Medicine, Texas A&M University, College Station, Texas 77843, United States

## Abstract

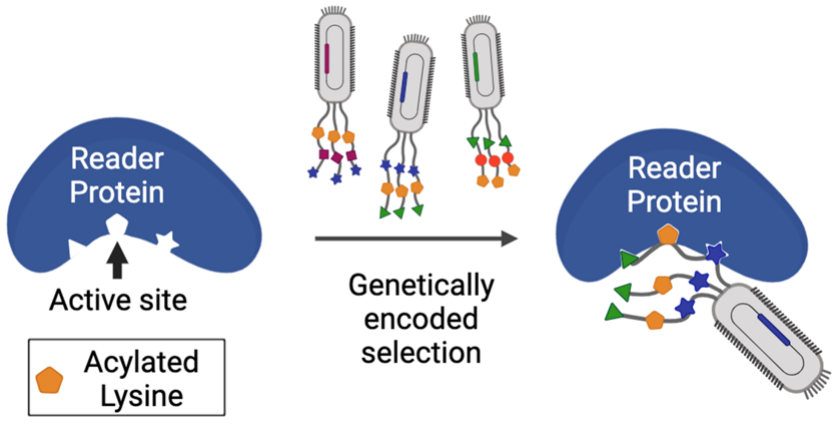

Epigenetic reader proteins interpret histone epigenetic marks to regulate gene expression. Given their vital roles and the link between their dysfunction and various diseases, these proteins present compelling targets for therapeutic interventions. Nevertheless, designing selective inhibitors for these proteins poses significant challenges, primarily due to their unique properties such as shallow binding sites and similarities with homologous proteins. To overcome these challenges, we propose an innovative strategy that uses phage display with a genetically encoded noncanonical amino acid (ncAA) containing an epigenetic mark. This ncAA guides binding to the reader protein’s active site, allowing the identification of peptide inhibitors with enhanced affinity and selectivity. In this study, we demonstrate this novel approach’s effectiveness by identifying potent inhibitors for the ENL YEATS domain that plays a critical role in leukemogenesis. Our strategy involved genetically incorporating *N*^*ε*^-butyryl-l-lysine (BuK), known for its binding to ENL YEATS, into a phage display library for enriching the pool of potent inhibitors. One resultant hit was further optimized by substituting BuK with other pharmacophores to exploit a unique π–π–π stacking interaction with ENL YEATS. This led to the creation of selective ENL YEATS inhibitors with a *K*_D_ value of 2.0 nM and a selectivity 28 times higher for ENL YEATS than its close homologue AF9 YEATS. One such inhibitor, tENL-S1f, demonstrated robust cellular target engagement and on-target effects to inhibit leukemia cell growth and suppress the expression of ENL target genes. As a pioneering study, this work opens up extensive avenues for the development of potent and selective peptidyl inhibitors for a broad spectrum of epigenetic reader proteins.

## Abstract 2

A genetically encoded acyl-lysine in phage-displayed peptides guides the rapid selection and identification of ultrapotent peptide inhibitors targeting an epigenetic reader protein that naturally binds the acyl-lysine.

## Introduction

Epigenetic reader proteins play a vital role in the regulation of gene expression and DNA repair.^1^ They function by interpreting, or “reading”, the various epigenetic marks such as methylation or acetylation added to histones. Reader proteins are diverse, varying in their specificity and affinity for different modifications and histone sequences.^2,3^ Dysregulation or malfunction of reader proteins can lead to erroneous interpretation of epigenetic marks, contributing to conditions such as cancer, neurological disorders, and cardiovascular diseases.^4,5^ Targeting these proteins offers a promising avenue for therapeutic intervention. The prospect of modulating the activity of epigenetic readers for therapeutic purposes has galvanized a significant amount of research in the field of drug discovery. One prominent example is the development of JQ1, a potent inhibitor of the BET family of bromodomains.^6^ However, the path to developing selective inhibitors for epigenetic readers is challenging. There is significant diversity among reader proteins, with each varying in their specificity and affinity for different modifications and histone sequences. This complexity makes it challenging to design small molecules that can selectively target and modulate a specific reader protein. Reader proteins often have shallow, featureless binding sites, making it harder for small-molecule drugs to bind with high affinity.^1^ Most epigenetic readers also share sequence and structure similarity with homologous proteins, making selective targeting of a particular reader protein using a small molecule difficult. As an example, JQ1 potently inhibits a group of BET family bromodomain proteins including BRD2, BRD3, BRD4, and the testis-specific protein BRDT.^7^

**Figure 1:**
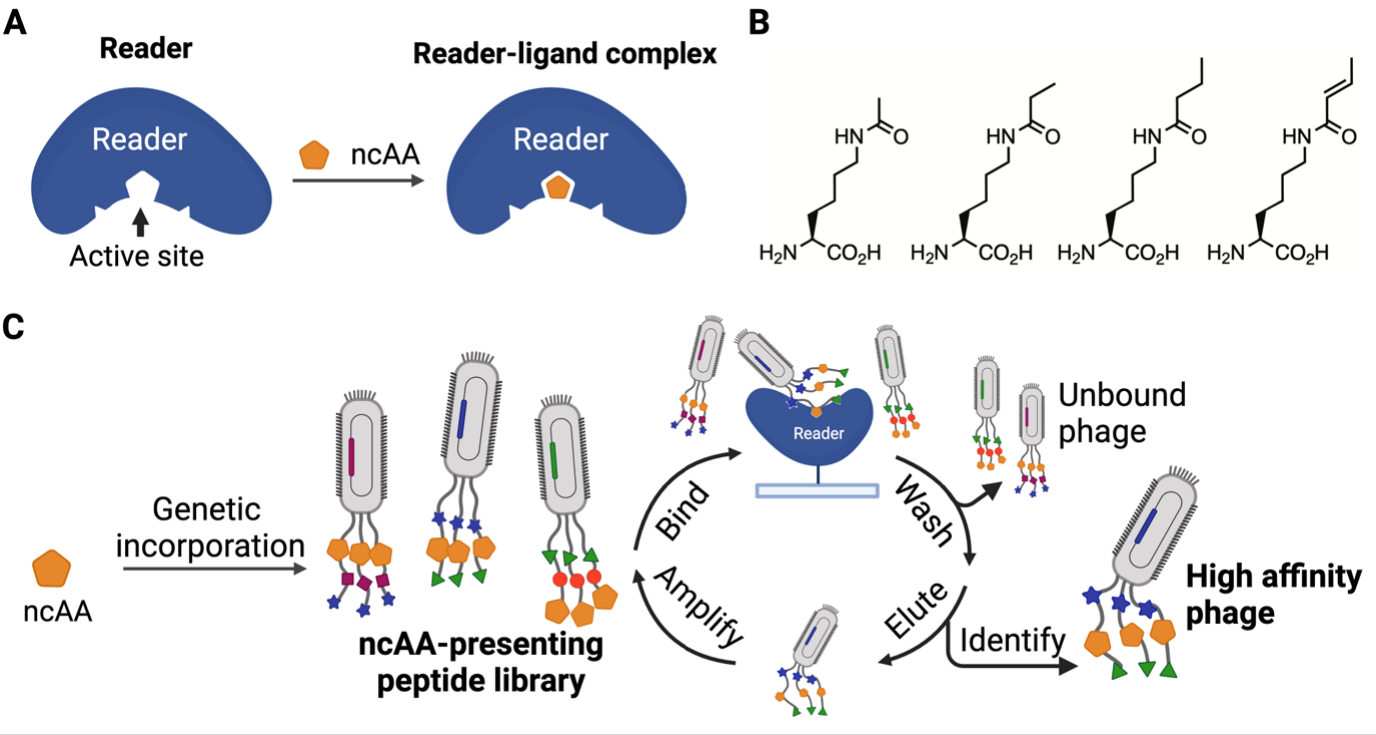
Schematic diagram of a genetically encoded noncanonical amino acid (ncAA) in phage display in guiding the selection of potent and selective peptide inhibitors for an epigenetic reader. (A) A diagram that illustrates the interactions between a reader protein and an epigenetic mark-containing ncAA. (B) Structures of four example ncAAs with epigenetic acylation marks that have been genetically encoded using PylRS mutants. (C) The genetic incorporation of an epigenetic mark-containing ncAA into phage-displayed peptides for active site-directed binding to an epigenetic reader that is followed by stringent wash and elution to select high-affinity and selective phages. Figure created with BioRender.com.

Despite the complexities involved, the pursuit of therapeutic strategies targeting epigenetic readers holds exciting potential.^8^ Realizing this potential is contingent on devising innovative strategies for drug development. Apart from utilizing its active site, referred to as the reader channel hereafter, to bind an epigenetic mark, a reader protein employs a shallow interaction area surrounding the reader channel to recognize a target protein. Therefore, a molecule capable of recognizing both the reader channel and its surrounding interaction area will likely achieve selective inhibition over similar proteins. This may require a shift from traditional small molecule approaches. In contrast to antibodies, which often face significant cellular permeability issues, peptides hold promise. They possess the potential of harnessing the extensive interaction interface like antibodies for improved binding, while also mimicking small molecules’ capacity for cellular permeability. The use of peptide display techniques, such as phage display, can facilitate high-throughput selection of peptides capable of potently binding to a reader protein. One promising strategy to prevent the selection of peptides not targeting the reader channel involves grafting the phage-displayed peptides with a chemical entity that acts as a binding anchor to the reader channel, thereby directing the selection process. We believe the incorporation of a genetically encoded noncanonical amino acid (ncAA) into phage displayed peptides,^9−12^ which selectively binds the reader channel, will fulfill all the requirements needed to identify a potent and selective inhibitor for an epigenetic reader (Figure 1). Previously, our team and others have developed the pyrrolysyl-tRNA synthetase (PylRS) alongside tRNA^Pyl^ to genetically incorporate many posttranslationally modified lysines, such as the acyl-lysines shown in Figure 1B and methyl-lysines.^13−18^ As these ncAAs are epigenetic marks, their amber-encoded incorporation into phage-displayed peptides should allow for the directed selection of potent and selective peptide inhibitors for an epigenetic reader. By constructing and selecting ncAA-encoding phage display libraries, it will be possible to identify potent peptide inhibitors for an epigenetic reader that use both the ncAA to target the reader channel and the amino acid residues flanking the ncAA to engage the surrounding interaction area, achieving both high potency and selectivity.

In this study, our objective is to illustrate the development and application of this innovative and unprecedented phage display technique in identifying potent and selective inhibitors for an epigenetic reader.

## Results and Discussion

### The ENL YEATS Domain as a Testing Model

To validate our proposed approach in the discovery of potent inhibitors for an epigenetic reader, we set our focus on the ENL YEATS domain as a testing model. YEATS domains are newly discovered epigenetic readers.^19,20^ In humans, four YEATS domain-containing proteins (ENL, AF9, YEATS2, and GAS41) bind to *N*^ε^-acetyl-l-lysine (AcK, lysine acetylation) in chromatin for taking part in transcription elongation, histone modification, and chromatin remodeling.^20,21^ Dysregulation of YEATS proteins has been linked to the onset and progression of cancers.^22−25^ In *mixed-lineage leukemia* rearranged leukemias (MLL-r leukemias), the *MLL1* gene (MLL) located on chromosome 11q23 is translocated and fused with partner genes including *MLLT1* (ENL) and *MLLT3* (AF9).^26−28^ The resultant chimeric proteins MLL-ENL and MLL-AF9 bind to several multisubunit complexes involved in transcriptional activation.^29−31^ A common mechanism of MLL-r leukemogenesis is mediated by AF9/ENL either through the reader function of YEATS domains or MLL fusions, suggesting a putative therapeutic window for AF9/ENL YEATS inhibitors in MLL-r leukemias. Despite their high sequence homology, the two close YEATS proteins AF9 and ENL appear also to play different roles in cancers. For example, it was established that ENL, but not AF9, is required for acute leukemia progression.^22,23^ In addition, knockout studies showed that depletion of ENL had minimal effects on normal hematopoietic stem cells.^23^ These findings suggest that selective inhibition of ENL could be an effective and low-toxicity approach to treat leukemia.

To date, a common rationale for the AF9/ENL inhibitor design exploits the tunnel-like property of the reader pocket. The AcK-binding pocket of YEATS domains shows an “end-open” characteristic, which is ideal to accommodate extended lysine acylation chains, such as *N*^ε^-propionyl-l-lysine (PrK, lysine propionylation), *N*^ε^*-*butyryl-l-lysine (BuK, lysine butyrylation), and *N*^ε^*-*crotonyl-l-lysine (CrK, lysine crotonylation).^32−36^ Crystallography studies have also revealed that AF9 and ENL bind preferentially to CrK over AcK via a π–π–π sandwich interaction involving two highly conserved aromatic side chains of F59 and Y78 and a CH-π interaction involving F28.^32,33,37^ The enhanced binding affinity is proposed to be attributed to the extra intermolecular force provided by the conjugated π system on the crotonyl group.^32,33^ These findings have led to the design and identification of a set of small molecule and peptidyl YEATS inhibitors.^36,38−43^ However, as the ligand-binding residues in reader pockets of AF9 and ENL are identical, it is difficult for an inhibitor to differentiate between the two solely by targeting the reader pocket alone. Molecules that display improved affinity and selectivity to either ENL or AF9 will likely need to provide extra interactions with the surrounding area of the reader pocket. A showcase of this type of molecules is JYX-3, an AF9 peptide inhibitor that targets a proximal site outside the reader pocket with a 30-fold selectivity for AF9 over ENL.^43^ JYX-3 was developed based on an AF9/ENL natural ligand K3K9cr in conjunction with a structure–activity relationship campaign.^42,43^ Since both BuK and CrK can be genetically incorporated into phage-displayed peptides,^10,15^ we believe a phage display library with BuK or CrK incorporated will allow us to easily fish out selective inhibitors for ENL by conducting selection against the ENL YEATS domain.

### Construction of a BuK-Containing Phage Display Library, Its Biopanning against the ENL YEATS Domain, and Characterization of Two Enriched Inhibitors

Ideally, CrK could serve as a suitable ligand for the ENL YEATS domain. However, in previous research, we and others discovered that when an electrophilic ncAA (e.g., a Michael acceptor) is present along with a cysteine in the phage displayed peptide, it will undergo proximity-driven cyclization that will complicate the selection process.^12,44^ In addition to CrK, YEATS domains recognize several other histone acylation marks, due to the end-open feature of the reader pocket. It was reported that AF9 YEATS binds BuK with enhanced affinity compared to AcK.^32,33^ Although lacking a π system, the extended acyl chain provides extra binding affinity through a CH-π interaction with F28.^32,33^ Therefore, BuK was chosen as an alternative ncAA ligand for the ENL YEATS domain in our study. The incorporation of BuK in a phage displayed peptide has been described.^10^ Briefly, BuKRS, a BuK-tRNA synthetase evolved from MmPylRS, was used to produce a phage in which the gIII gene contained an amber codon in the *N*-terminal coding region. Titer results showed nearly 100-fold difference in phage production in response to the absence and presence of BuK, demonstrating the successful incorporation of BuK into phage-displayed peptides (Figure S1).^10^

**Figure 2:**
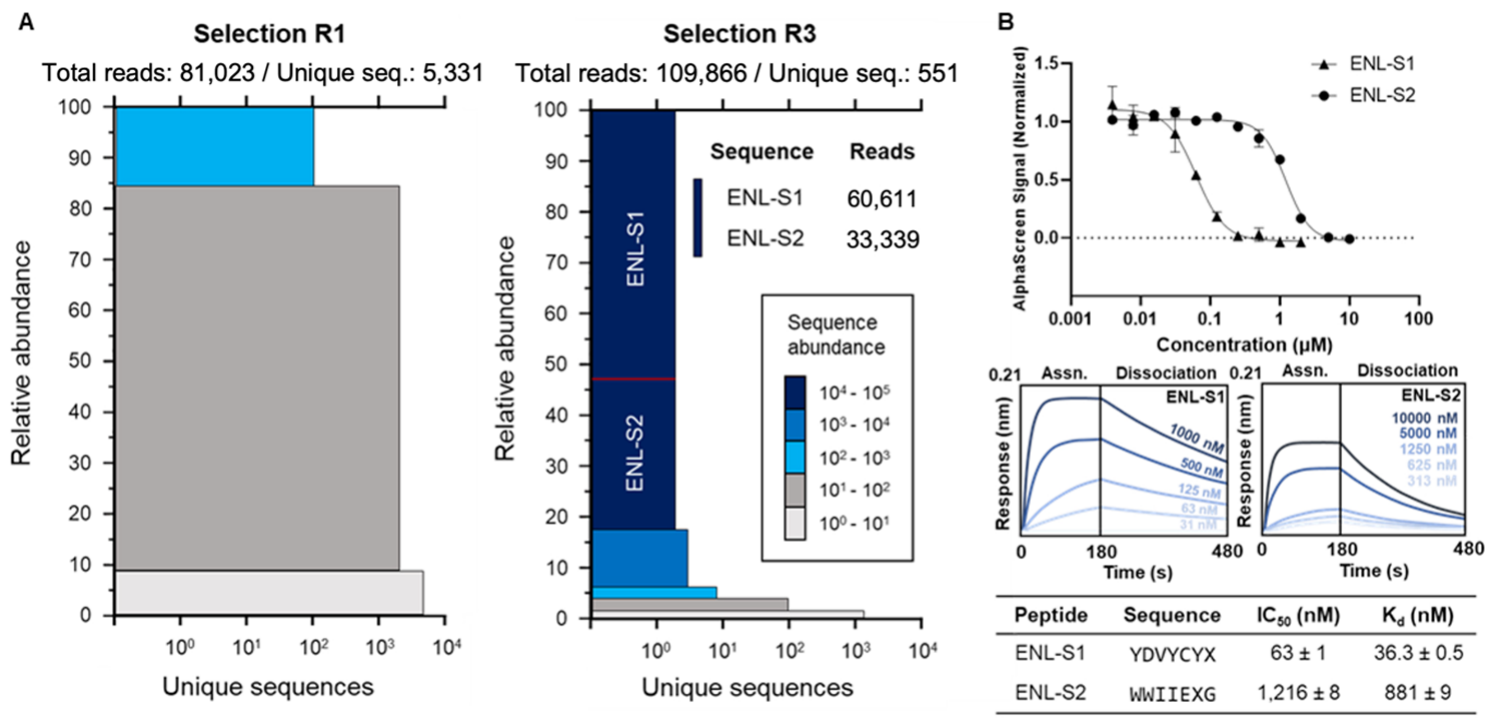
Phage panning identified two peptide ligands for ENL YEATS. (A) The visualization of next-generation sequencing results from first and third panning rounds. Each colored block represents all sequences with the specific abundance. The block height corresponds to the percent abundance of sequences in each sector, and the width represents the number of unique sequences per sector. After three rounds of panning, the library converged into two highly enriched sequences, **ENLS1** (YDVYCYX) and **ENLS2** (WWIIEXG). X denotes BuK. (B) Binding and inhibition parameters of the selected peptides. Binding kinetics are characterized by BLI with varying concentrations of selected peptides. The nonlinear least-squares-fitting curves are represented. IC_50_ values are measured with AlphaScreen assays and given as the mean ± standard deviation (SD) of three individual experiments (*n* = 3).

To apply the active site-directed ligand search for the ENL YEATS domain, the BuK-presenting peptides (7-mer) were displayed on phage using the method previously developed.^10^ We utilized a previously established amber-obligate 7-mer phage display library to develop a phage display library enriched with BuK-containing 7-mer peptides. For detailed characterizations, including analysis of amber codon percentages, NNK trinucleotide analysis, and amino acid distribution at each position within the library, please refer to the work of Tharp et al.^10^ Furthermore, the comprehensive raw NGS data for the amber-obligate 7-mer phage library can be found in the NCBI’s Sequence Read Archive, under the accession ID PRJNA606283. To produce the BuK-containing 7-mer phage library, the phages were produced in the presence of 5 mM BuK using TOP10 cells harboring the following three plasmids: (1) pADL-gIII, which contains the 7-mer amber-obligate peptide-coding DNA library positioned at the *N*-terminus of M13 pIII gene; (2) pCDF-BuKRS, which encodes BuKRS and its cognate tRNA^Pyl^ for the genetic incorporation of BuK at amber codons in the 7-mer library; and (3) M13KO7TAA, a helper phage plasmid engineered to have a TAA mutation at pIII so that its lack of a functional pIII allows an efficient multivalent display of the 7-mer library. The ENL YEATS domain containing an *N*-terminal AviSUMO-Tag was recombinantly expressed in *E. coli* together with BirA, a biotin ligase, for site-specific biotinylation at the AviTag. The biotinylated AviSUMO-ENL YEATS was immobilized on streptavidin-coated magnetic beads for panning with the BuK-containing phages. The selection pressure was gradually increased by lowering the protein usage and increasing the number of washes to promote enrichment of potent binders. Through three rounds of panning, an approximately 362-fold increase in the total number of eluted phages was achieved in spite of the increased selection pressures, suggesting a successful selection (Figure S5). Phage libraries collected from the first and third rounds of selection were sequenced using Illumina next generation sequencing (NGS) and all sequenced data are available through the NCBI SRA database (Project PRJNA1041845). To prevent possible sequencing errors from affecting conclusions, sequences were filtered using previously published paired-end processing,^10^ where any sequences containing more than one mismatch in the nonlibrary region or any mismatch in the library region were discarded. The NGS data provided DNA sequences encompassing both the library and nonlibrary regions. In our analysis, we included data from the nonlibrary region as a stringent quality control measure to filter out low-quality data. For our analysis, we utilized 51 nucleotides from the NGS output, with 21 nucleotides encoding the library region and an additional 30 nucleotides for the nonlibrary region. We permitted a single mismatch within the nonlibrary region to preserve data breadth without compromising the quality of data analysis, as mismatches in this region do not affect the integrity of the library sequence data. The deep sequencing results from 81,023 filtered reads showed that 5,331 unique sequences were obtained from the first round of selection (Figure 2A). Among all the sequences, only 85 of them contained more than 100 copies (blue area), while the remaining sequences all had low abundances (<10^2^ copies, dark and light gray areas), which constituted 85.2% of the first round population. After three panning rounds, the selection converged to two highly enriched sequences: YDVYCYX (**ENL-S1**) and WWIIEXG (**ENL-S2**, X denotes BuK). Out of 551 different unique sequences collected from the third round, **ENL-S1** and **ENL-S2** represented 85% of the entire library (Figure 2A and Figures S6–S8). Both peptide sequences corresponded to their own unique DNA sequence, rather than there being varying codons encoding the same amino acids (Figure S7). This indicates that they were all enriched from the same two original clones. Two motifs were found in the majority of sequences with high abundance (greater than 0.1%) (Figure S6). A Y(C/L)Y pattern was identified in the residues adjacent to BuK in four of the eight top sequences. Also, an H(L/Y)(T/V)LFXG (X denotes BuK) motif was observed, albeit at lower abundances, in two of the top sequences. In the initial round of selection, we observed that only a small percentage of phages were eluted, which aligns with typical outcomes in phage selection experiments since only a low copy number of phages can bind to the target.^10,45^ With each successive round of selection, we noted a gradual increase in the percentage of phages eluted. This incremental, rather than sharp, increase was attributed to the intensification of selection pressures, such as the application of more stringent washing protocols. We aimed for the incremental increase to retain potent clones with low copy numbers from the previous selection cycle. By enhancing the wash stringency in each round, we sought to prevent the predominance of high copy number clones with comparative low potency in the final eluted population. To validate the necessity of BuK for selectivity during biopanning, a control selection was performed using the same library but in amber-suppressing ER2738 cells that incorporate glutamine at amber codons. Input titers for the negative selection were higher than the positive selection using phages containing BuK, owing to the phage expression in ER2738 cells being less affected by amber suppression than in cells coding BuK. Although phage titers indicated increased phage binding after three rounds of selection against ENL YEATS, the percent of phages binding to the protein was consistently lower compared to the BuK selection (Figure S9). The observation of phage titer increase in the control selection process was anticipated since certain peptides on non-BuK-encoding phage would still bind the ENL YEATS domain. Illumina sequencing indicated little enrichment of the control library in comparison to the library containing BuK, with the most abundant sequences showing negative enrichment between rounds 1 and 3 (Figures S9–S11). In addition, there was a reduced trend toward consensus sequences in the negative selection process. Given the significant variation often between titer values, sequencing data often offers a more dependable measure of selection success. That was what we observed for BuK-encoding phage but not non-BuK-encoding phage. Given these results, we concluded that BuK was necessary for an effective selection strategy, and we looked to characterize ENL-S1 and ENL-S2 for inhibition of ENL YEATS.

To test the inhibition potency of the two selected peptides for the ENL YEATS domain, **ENL-S1** and **ENL-S2** were chemically synthesized through solid-phase peptide synthesis (SPPS) and evaluated by an AlphaScreen assay. In this assay, a biotinylated H3K27cr peptide and a 6xHis-tagged ENL YEATS domain (His-ENL YEATS) were immobilized on PerkinElmer streptavidin donor and nickel-chelating (Ni-NTA) acceptor beads, respectively. The use of a His-ENL YEATS instead of the original AviSUMO-ENL YEATS construct in the assay prevents potential false positive results derived from ligands which bind to the SUMO protein but not to the ENL YEATS domain. Results in Figure 2B showed that **ENL-S1** and **ENL-S2** inhibited interactions between ENL YEATS and H3K27cr with an IC_50_ value of 63 ± 1 and 1,216 ± 8 nM, respectively. We then assessed the binding affinity of two ligands for the ENL YEATS domain using biolayer interferometry (BLI). Briefly, His-ENL was immobilized onto Ni-NTA functionalized biosensors to measure the binding kinetics of target peptides at different concentrations. Results showed **ENL-S1** binds strongly to the ENL YEATS domain with a determined dissociation constant *K*_D_ of 36.3 ± 0.5 nM, whereas **ENL-S2** binds more than 20-fold weaker to the target protein with a *K*_D_ value of 881 ± 9 nM (Figure 2B and Figure S12). With a 36 nM *K*_D_ value, **ENL-S1** represents one of the most potent inhibitors that has been developed for the ENL-YEATS domain. As a small peptide directly selected from a phage display library, this level of high potency was unexpected. Potent binders with nanomolar and even picomolar *K*_D_ values are frequently selected from phage-displayed ScFv and nanobody libraries. However, shorter-than-10-mer linear peptides with nanomolar binding potency have rarely been directly selected from phage display libraries. One example was S2P03, a 7-mer peptide that has a *K*_D_ value as 49 ± 9 nM to bind to SIRT2.^10^ This peptide contained BuK and was identified also from a BuK-containing phage display library using the active site-directed ligand selection technique. Taken together, the two examples confirmed that utilizing phage display libraries incorporated with a ncAA serving as a ligand for a protein target for active site-directed ligand selection is a successful method for identifying ultrahigh potency ligands for epigenetic proteins including both enzymes and readers. For **ENL-S1** and **ENL-S2**, their determined *k*_on_ and *k*_off_ values were 6.23 ± 0.07 × 10^4^ M^–1^ s^–1^, 6.29 ± 0.06 × 10^3^ s^–1^ and 2.26 ± 0.02 × 10^–3^ M^–1^ s^–1^, 5.54 ± 0.02 × 10^–3^ s^–1^, respectively. Compared to **ENL-S2**, **ENL-S1** has faster binding to the ENL YEATS domain and slower release from it, indicating its potential long-term action on the ENL YEATS inhibition. Collectively, our data confirmed that **ENL-S1** displayed much better potency than **ENL-S2**, making it an ideal candidate for further optimization as a high-affinity ENL inhibitor.

### Optimization of ENL-S1 with Alternative Reader Channel Binders and for Potential Cellular Permeability

**Figure 3:**
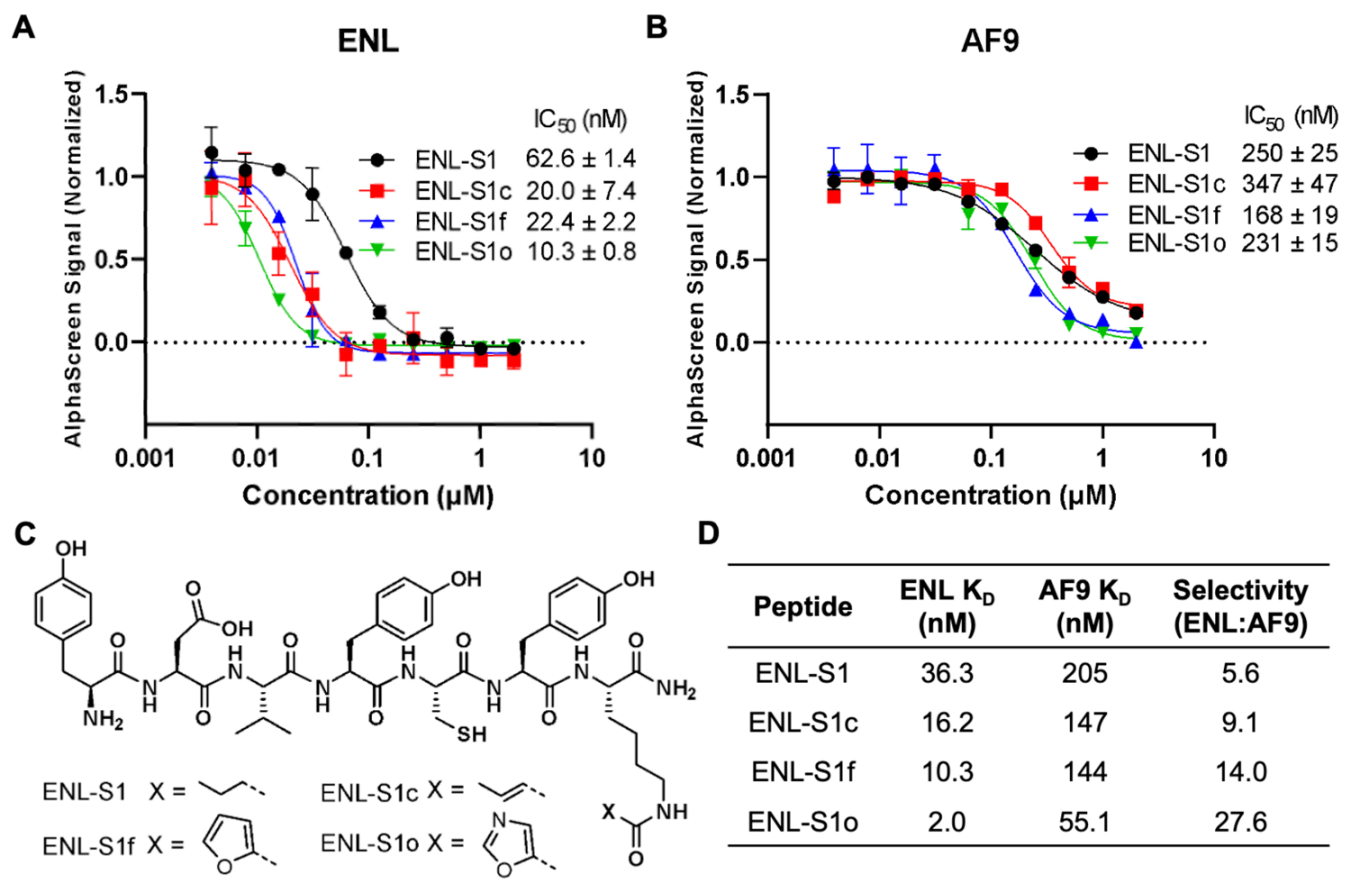
Characterization and optimization of **ENL-S1**. (A, B) AlphaScreen analysis of **ENL-S1** and its derivatives in the inhibition of (A) ENL and (B) AF9 YEATS binding to their corresponding acylated histone peptides. IC_50_ values are given as the mean ± SD, *n* = 3. (C) Chemical structures of **ENL-S1** and three derivatives developed to cultivate the π–π–π stacking interaction with ENL YEATS. (D) BLI-determined binding affinities of **ENL-S1** and its derivatives for AF9 and ENL YEATS.

AF9 and ENL YEATS domains bind preferentially to CrK over AcK via a π–π–π sandwich interaction with two aromatic residues F59 and Y78 in the YEATS domain (positions correspond to that in the ENL YEATS domain).^32,33^ Since the π–π interactions are not available to the butyryl group, we envisioned that replacing the BuK residue in **ENL-S1** with a conjugated π system will lead to a more potent inhibitor through the enhanced stacking interactions. For this reason, we generated **ENL-S1c** that has CrK in the original BuK position. In previous research, two modified H3 peptides with *N*^ε^*-*(furan-2-carbonyl)-l-lysine (FurK) and *N*^ε^*-*5-oxazolecarbonyl-l-lysine (OxaK) that contained expanded π systems to provide extra intermolecular interactions with the AF9 YEATS domain showed a 4.5- and 14.7-fold binding enhancement compared to that with CrK.^42^ Inspired by this finding, we synthesized **ENL-S1f** and **ENL-S1o** with FurK and OxaK replacing BuK in **ENL-S1** (Figure 3C). As predicted, all three new compounds showed increased potency compared to **ENL-S1** with IC_50_ values as 20, 22, and 10 nM, respectively (Figure 3A), supporting the role of a conjugated π system in achieving strong binding to a YEATS domain. Notably, the correlation between substituents and binding affinity also suggests that BuK in **ENL-S1** is positioned in the π–π–π sandwich cage of ENL YEATS to guide displayed peptides to the active site. All three inhibitors were also characterized using BLI. They all have determined *K*_D_ values below 20 nM (Figure S13). Among them, **ENL-S1o** has a remarkably low *K*_D_ value of 2.0 nM. Binding affinity at this level is equivalent to that for most therapeutic antibodies for their targeted antigens. As far as we know, this is the strongest inhibitor that has been developed for the ENL YEATS domain. All three inhibitors displayed fast binding to ENL YEATS and slow release from it, indicating their potential long-term action for ENL YEATS inhibition. To further confirm the affinity of the peptides for ENL, we developed an AlphaScreen assay by synthesizing a biotin conjugated ENL-S1 (Biotin-ENL-S1) and tested for its ability to interact with His-ENL using Ni-NTA and Streptavidin Alpha beads. The observed *K*_D_ was 72 ± 7 nM, which validated the ability for the selected peptides to bind in the low nanomolar range (Figure S14). We hypothesize the slightly lower affinity in comparison to the unlabeled peptide may be due to modification of the *N*-terminus with the biotin.

These potent inhibitors were then tested to confirm their selectivity for ENL YEATS over AF9 YEATS since the two YEATS domains share high sequence and structure homology. One potential of using ncAA-containing phage display for active site-directed ligand search is the possibility of amino acids flanking the ncAA in a selected peptide to selectively engage the target over a close homologue. We wanted to validate this potential as well. Selective targeting of ENL YEATS is also preferred in leukemia interventions as it plays a pivotal role in the progression and maintenance of several AMLs and poses minimal harm to normal hematopoietic stem cells upon inhibition. The inhibition assays performed with the AF9 YEATS domain showed that **ENL-S1** and all three derivatives are more selective toward ENL compared to AF9 YEATS (Figure 3B). Judging from IC_50_ values, **ENL-S1**, **ENL-S1c**, **ENL-S1f**, and **ENL-S1o** displayed 4.0-, 18-, 7.7-, and 23-fold lower potencies toward AF9 YEATS compared to ENL YEATS. These results show that despite the high sequence and structure similarity between AF9 and ENL YEATS domains, our identified peptides can distinguish between these two highly conserved domains. We propose that the selectivity arises from the peptide residues interacting with surface areas adjacent to the active site of ENL YEATS. These results showcase the practicality of the active site-directed ligand selection from phage display to develop potent and selective inhibitors. Similarly, BLI analyses were conducted to examine preferential binding of inhibitors to ENL YEATS. In agreement with the results from the inhibition assays, all four peptides showed selectivity toward ENL YEATS over AF9 YEATS (Figure 3D and Figure S13). In particular, **ENL-S1** (*K*_D,ENL_ = 36.3 nM, *K*_D,AF9_ = 205 nM) and **ENL-S1c** (*K*_D,ENL_ = 16.2 nM, *K*_D,AF9_ = 147 nM) displayed 5.6- and 9.1-fold higher affinities for ENL YEATS. Two peptides with extended π systems, **ENL-S1f** (*K*_D,ENL_ = 10.3 nM, *K*_D,AF9_ = 144 nM) and **ENL-S1o** (*K*_D,ENL_ = 2.0 nM, *K*_D,AF9_ = 55.1 nM), showed even higher preference for targeting ENL YEATS, as they bound with 14.0- and 27.6-fold, respectively, stronger affinity for ENL than AF9. Overall, these data demonstrate that our peptides preferentially target ENL over AF9 and confirm that active site-directed ligand selection from ncAA-containing phages is a practical technique for the identification of selective peptide inhibitors for an epigenetic reader. Two previous publications have documented peptide-based YEATS inhibitors featuring alkylated lysine residues.^42,43^ Notably, the most effective peptide inhibitor reported in these studies exhibited an IC_50_ value for the AF9 YEATS domain at 240 nM, where almost all peptides reported showed IC50 values exceeding 1 μM for the ENL YEATS domain. With peptides developed in our current work exhibiting over 100-fold increase in potency compared to previously reported peptides, we are confident that our ncAA-directed, phage-assisted methodology is a highly effective strategy for identifying potent inhibitors of epigenetic readers.

**Figure 4:**
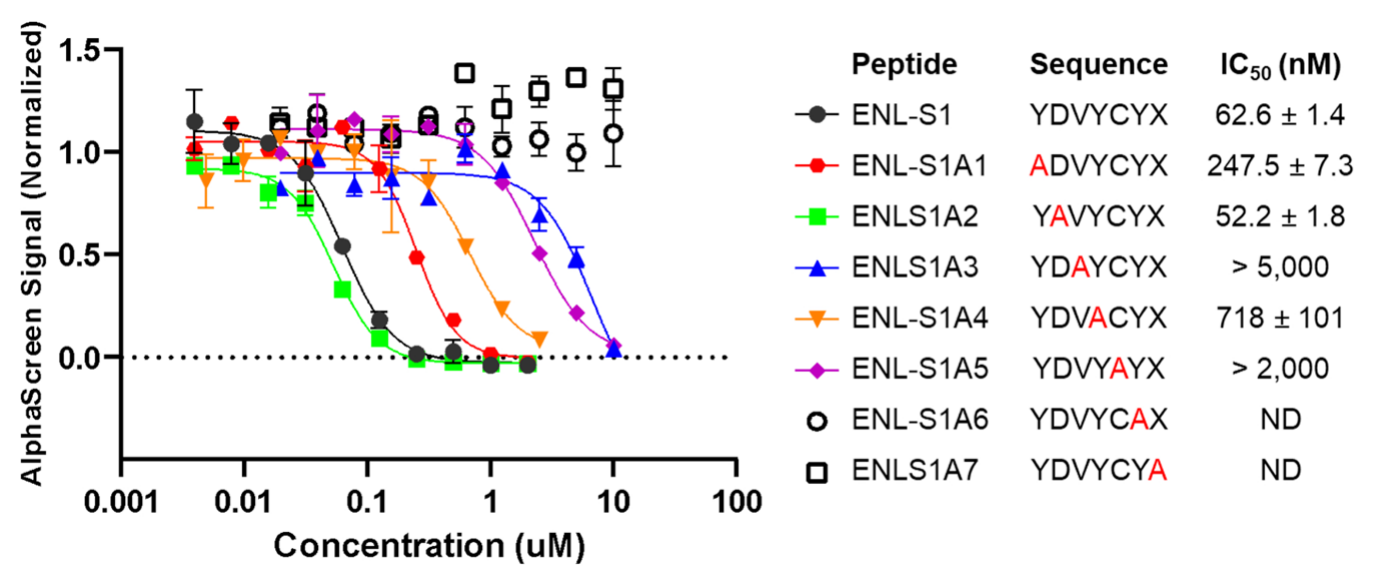
Structure–activity relationship studies of **ENL-S1** to identify key residues. An alanine scan was performed by iteratively replacing each residue with an alanine and testing for change of ENL inhibition through AlphaScreen assays. IC_50_ values are given as the mean ± SD, *n* = 3.

**Figure 5:**
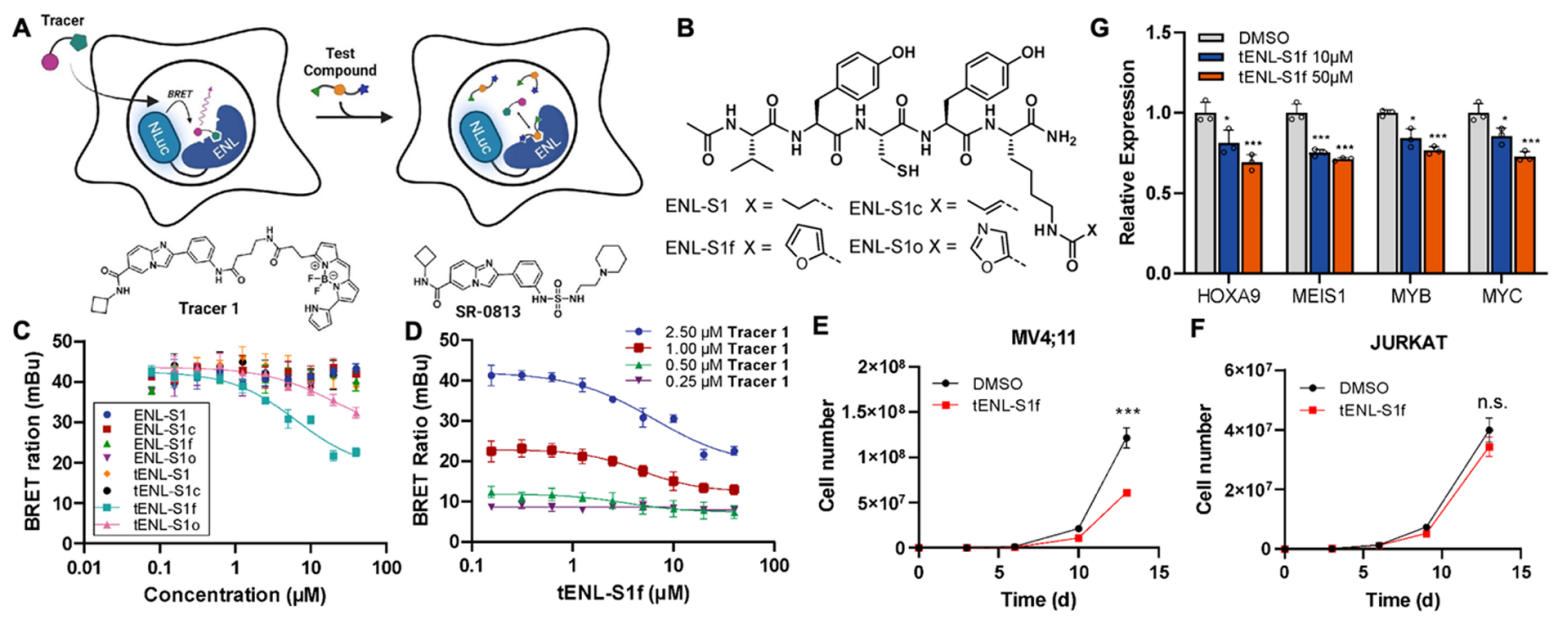
(A–D) Development of a NanoBRET assay for the cellular target engagement profiling of ENL inhibitors. (A) A schematic illustration of the NanoBRET assay design for ENL YEATS. A NanoLuc luciferase (NLuc)-ENL YEATS fusion protein is expressed in HEK293T cells. **SR-0813T** (bottom left) developed from an ENL inhibitor SR-0813 (bottom right) is used to achieve BRET. Upon **SR-0813T** binding to ENL YEATS, luminescence generated from NLuc is transferred to the tracer fluorophore, resulting in a fluorescence emission. A test compound will competes with **SR-0813T** for binding to ENL YEATS, leading to an attenuated fluorescence signal. (B) Chemical structures of truncated **ENL-S1** derivatives. (C) NanoBRET curves of **ENL-S1** and its derivatives displaying their relative affinities for ENL YEATS expressed in HEK293T cells. (D) Measurement of the apparent intracellular affinity of **tENL-S1f** for ENL-YEATS. (**E-G**) On-target effects of **tENL-S1f** in leukemia. Cell growth inhibition by **tENL-S1f** for (E) MV4;11, an ENL-dependent MLL-r leukemia cell line and (F) JURKAT, an ENL-insensitive acute leukemia cell line. (G) qPCR analysis of transcript abundance in MOLM13 cells treated with **tENL-S1f** (normalized against a DMSO control). Raw BRET ratios in panels C and D are shown as mean ± SD, *n* = 3. Data in panels E–G represent Mean ± SD, *n* = 3, and *P* values by two-tailed Student’s *t* test against a DMSO control. *P < 0.05, **P < 0.01, ***P < 0.001. Not significant (n.s.) *P* > 0.05. The schematic representation shown in panel A was created with BioRender.com.

Although **ENL-S1** and its derivatives showed promising results in *in vitro* assays, their 7-mer peptide nature leads to a concern about their access to the nucleus, where ENL is localized. Therefore, to improve the physicochemical properties of **ENL-S1**, an alanine scan was performed to study its SAR (Figure 4 and Figure S15). The analysis showed that replacing the second residue with alanine slightly improved inhibition. Replacing the two tyrosine residues at the first and fourth positions resulted in a 4–11-fold decrease in inhibition. Mutation of the third (Val) and fifth (Cys) residues caused dramatic decreases (>30-fold) in IC_50_, suggesting these residues are crucial for ENL binding affinity. As predicted, replacing BuK with alanine abolished the inhibition, confirming its vital role in the molecular recognition. Interestingly, substituting the sixth residue also resulted in complete loss of inhibitory activity, indicating this tyrosine is critical for the binding to ENL YEATS. Since the alanine scan indicated that the replacement of first tyrosine and second aspartic acid resulted in modest changes in inhibition, we removed the first two amino acids while retaining the rest of the essential residues. In addition, an acetyl protecting group was added to the *N*-terminus to prevent *N*-terminal degradation and to increase peptide permeability. Subsequently, four inhibitors (**tENL-S1**, **tENL-S1c**, **tENL-S1f**, and **tENL-S1o**) were generated for further characterization (Figure 5B).

### Validation of ENL YEATS Inhibitors by NanoBRET Assays and the Antitumor Potency of tENL-S1f

For peptides developed for intracellular targets, a common concern is their cellular permeability to engage the targets. For the ENL YEATS domain that is a nuclear protein, its inhibitors will also need to translocate to the nucleus to exert their inhibition activities. Before we fully assessed all developed peptides, we did a pilot study to analyze the cell penetration property of tENL-S1o that was the most potent truncated peptide. We attached FITC to tENL-S1o to afford FITC-tENL-S1o that could be fluorescently imaged. When this molecule was incubated with MV4-11 and MOLM-13 cells, strong fluorescence within the cells was detected just 2 h post incubation (Figure S17). Although results from this pilot study were very encouraging, the conjugated FITC could have assisted the cellular penetration process. A more accurate assay was needed for the use of unmodified peptides. In light of this, we proceeded to use a NanoBRET assay to evaluate whether our developed peptides could permeate into cells and engage the ENL YEATS domain in the cellular melieu.^46^ The NanoBRET assay is an emerging cutting-edge technique that is robust and sensitive in assessing not just cellular permeability of a developed ligand but also the ligand engagement with its protein target in the cellular environment. A significant advantage of the NanoBRET assay over the fluorophore conjugation and other approaches is allowing intact ligands to be used. No physical and chemical modifications to them are necessary. There are three components required to gauge the peptide inhibitory potency for ENL expressed in cells through NanoBRET assays: (1) an ENL YEATS-NanoLuc luciferase fusion protein, (2) a NanoLuc luciferase substrate, and (3) a fluorescent tracer that binds specifically and reversibly to the reader channel of ENL YEATS. In this assay, the substrate furimazine reacts with NanoLuc luciferase to produce bioluminescence, which is quenched via bioluminescence resonance energy transfer (BRET) by the nearby fluorescent tracer bound to the ENL YEATS-NanoLuc fusion. The cellular potency of peptides can be determined through the decrease in BRET as test peptides engage the ENL YEATS domain and displace the tracer. Previously, a NanoBRET assay based on histone 3.3 (H3.3) was developed for YEATS proteins.^38^ While it was successfully used to confirm the cellular activity of YEATS inhibitors against AF9, no responses were observed for ENL upon inhibitor treatment. This was likely attributed to the difference in binding affinities between AF9 and ENL to histone acetylation marks.^19,22^ Since the BRET signal is dependent on the ligand-protein association, having an inadequate interaction between histone H3.3 and ENLYEATS may result in a small assay window and poor applicability.

In light of this, we developed our NanoBRET assay based on **SR-0813T**,^47^ a small-molecule tracer adapted from a highly potent ENL inhibitor SR-0813 (Figure 5A).^40^**SR-0813T** was generated by appending a pyrrolyl substituted BODIPY (Py-BODIPY), which is relatively insensitive to solvent pH and polarity changes and lack of charges and molecular polarity for potential disruption of properties of tagged ligands,^48,49^ to the SR-0813 scaffold via a short linker, 4-aminobutyric acid. Meanwhile, we established a stable cell line for NanoLuc-ENL YEATS expression. The binding of **SR-0813T** to ENLYEATS was also validated using a fluorescence polarization assay (Figure S16). **SR-0813T**, compared to H3 peptides, exhibited an improved binding affinity for ENL YEATS (*K*_D_ = 1.1 μM), albeit weaker than its parental compound SR-0813, presumably due to the chemical modifications introduced by conjugation.

Using the developed ENL-NanoBRET assay, we then characterized the cellular potency of our developed peptidyl inhibitors. The analysis was done in the presence of 2.5 μM of **SR-0813T** and varying concentrations of inhibitors (Figure 5C). All linear 7-mer peptides showed no detectable inhibition against ENL YEATS within the assay window. Two of the four truncated **ENL-S1** derivatives exhibited ENL engagement; **tENL-S1f** showed a clear dose-dependent displacement of **SR-0813T** from NanoLuc-ENL (IC_50_ = 6.4 ± 1.6 μM), whereas **tENL-S1o** showed some cellular activity with ∼50% inhibition at 40 μM. The apparent intracellular affinity of **tENL-S1f** was further evaluated by NanoBRET at various tracer concentrations (Figure 5D). Overall, the peptide inhibited ENL with IC_50_ values in the low-micromolar range in cells (IC_50,0.5μM_ = 4.1 ± 1.1 μM, IC_50,1.0μM_ = 4.2 ± 1.1 μM, IC_50,2.5μM_ = 6.4 ± 1.6 μM). BLI analysis of **tENL-S1f** confirmed that the inhibitor is selective for ENL YEATS over three other human YEATS proteins with a 12.0-fold selectivity over AF9 and no binding to YEATS2 and GAS41 up to 10 μM **tENL-S1f** used (Figure S18). These results suggested that the truncation of the 7-mer peptide **ENL-S1f** to afford **tENL-S1f** allowed for cellular target engagement while retaining the selectivity toward the target, although at the cost of its potency. In our prior alanine scanning analysis, substituting the cysteine residue with alanine resulted in a marked reduction in **tENL-S1f**’s affinity for the ENL YEATS domain. This notable result prompted us to investigate the possibility of cysteine contributing to an active dimeric form. Nonetheless, rapid aggregation of the dimer prevented its use in subsequent analyses. Based on these results, we deduce that the monomeric, reduced state of tENL-S 1f constitutes its active form.

Encouraged by the cellular studies, we next examined the effects of **tENL-S1f** on acute leukemia proliferation in an MLL-r cell line MV4;11 (MLL-AF4 AML) whose growth is sensitive to ENL depletion.^23^ We found that **tENL-S1f** exhibited ∼50% inhibition of MV4;11 cell growth at 10 μM after 14 days of treatment (Figure 5E). In contrast, the growth pattern of JURKAT cells (T-cell ALL), an ENL-independent cell line,^23^ showed no detectable response to the treatment with **tENL-S1f** (Figure 5F). As aforementioned, ENL drives leukemogenesis through the ENL YEATS-chromatin association at oncogenic gene loci. To examine whether our peptide disrupts the ENL YEATS-chromatin interaction and thereby downregulates oncogene expression, we evaluated the relative mRNA level of several ENL-target genes, including *HOXA9*, *MEIS1*, *MYB*, and *MYC* in MOLM-13 cells (MLL-AF9 AML) treated with **tENL-S1f**. Quantitative PCR results showed all genes were suppressed in response to **tENL-S1f** treatment at 10 μM. At 50 μM, a ∼30% loss in transcription levels was observed for all four leukemic genes (Figure 5G). Altogether, these results indicate **tENL-S1f** exhibits on-target effects, suppresses ENL target gene expression, and ultimately inhibits ENL-dependent leukemia proliferation.

### MD Simulations to Predict the Interactions between tENL-S1f and ENL YEATS

**Figure 6:**
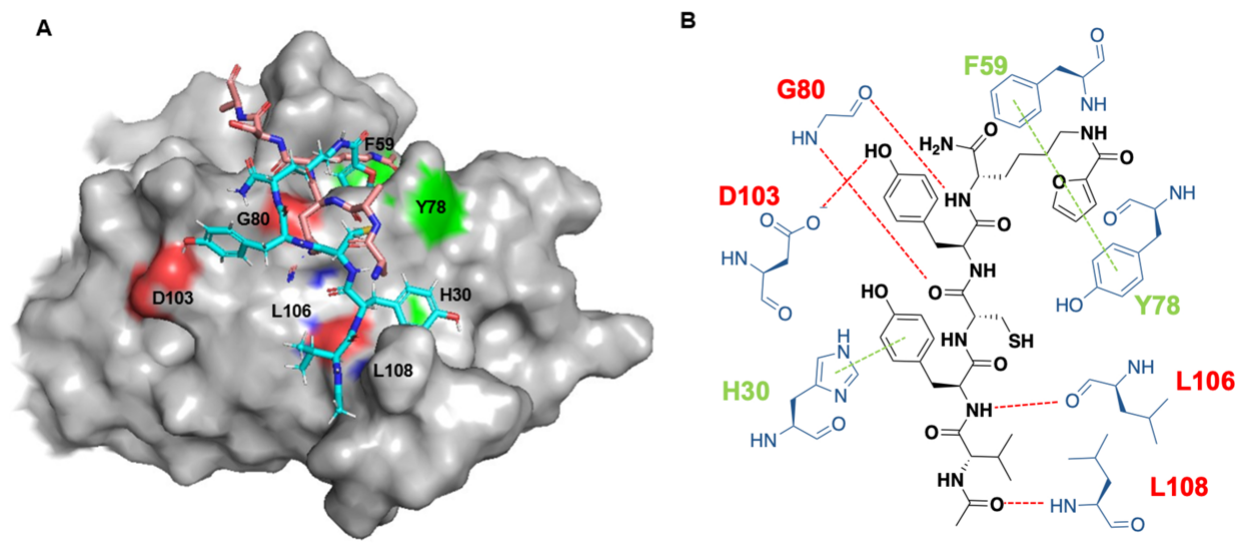
Molecular dynamic simulations to predict the interactions between **tENL-S1f** and ENL YEATS. (A) The MD simulation predicted complex structure of **tENL-S1** (cyan) bound to the substrate binding site of ENL YEATS (gray). The native substrate H3K27Ac peptide is shown in salmon. (B) Interactions between **tENL-S1f** and ENL YEATS predicted by molecular dynamic simulations. π-stacking (green) and hydrogen-bond (red) interactions are shown in dashed lines with distance indicated in angstroms. MD simulations were performed based on the reported crystal structure (pdb: 5J9S).

To elucidate the interaction between **tENL-S1f** and the ENL YEATS domain, we endeavored to obtain cocrystals of the complex. Unfortunately, the ENL YEATS domain exhibited rapid aggregation under our screening conditions, precluding the formation of analyzable crystals for X-ray crystallography. As an alternative approach, we performed the molecular dynamic (MD) simulation using a published ENL YEATS domain structure (PDB: 5J9S) as the starting model.^22^ The binding of **tENL-S1f** to the AF9 YEATS domain (PDB: 4TMP) was simulated as well as a control. In our simulations, we did not impose any initial constraints to compel the furan-2-carbonyl group’s binding within the reader channel. Nevertheless, both simulations naturally converged on an optimized binding model where the side chain of FurK was deeply embedded within the reader channel. In the AF9 simulation, the predicted structure showed **tENL-S1f** bound to both the reader channel and the peptide binding cleft of the AF9 YEATS domain and overlapping with the H3K9Ac peptide that was the native substrate of the AF9 YEATS domain (Figure S19).^19^ Particularly, F59 forms a π–π interaction reaction with the furan group in **tENL-S1f** and three residues (S58, Y78, and A79) engage three hydrogen bonds to interact with the side chain amide of FurK. G80 located at the end of the reader channel forms three hydrogen bonds, and L108 at the lower end of the substrate recognition region forms one hydrogen bond with the backbone of **tENL-S1f**. In comparison, our simulations indicated that **tENL-S1f** interacts with the ENL YEATS active site in a conformation distinct from that observed with AF9, as depicted in Figure 6A. The critical interactions are detailed in Figure 6B. Notably, two aromatic residues, F59 and Y78, establish a complex π–π–π stacking interaction with **tENL-S 1f**’s furan moiety. A similar interaction was not observed with AF9. The backbone amines and carbonyl oxygens of G80, L106, and L108 form a quartet of hydrogen bonds with **tENL-S1f**’s backbone. Remarkably, the side chain of H30 contributes an additional π–π stacking interaction with **tENL-S 1f** by engaging the aromatic side chain of Y2, underscoring a strong preference of Y2 at this position. Furthermore, D103’s side chain carboxylate forms a hydrogen bond with the phenolic hydroxyl group of Y4 in **tENL-S1f**, clarifying the necessity of Y at this position for high-affinity binding to the ENL YEATS domain. These interactions are absent in the simulated structure of the AF9 YEATS-**tENL-S1f** complex. We believe the distinct structural variations observed in the two simulated structures account for the specificity of **tENL-S1f** toward the ENL YEATS domain over the AF9 YEATS domain. In the modeled structure o ENL YEATS bound to **tENL-S1f**, the thiol group of the C3 side chain in **tENL-S1f** engages in hydrophobic interactions within the peptide binding groove of the ENL YEATS domain. The dual nature of cysteine to participate in both hydrophobic and hydrophilic interactions is well-established in the literature.^50^ This hydrophobic engagement of the C3 thiol group appears to be pivotal for binding affinity, as evidenced by a marked reduction in binding upon substituting C3 with alanine. One notable outcome from the simulations was the unexpected absence of interactions between the *N*-terminal valine and the ENL YEATS domain. This was surprising, given our prior observation that substituting this valine with alanine significantly decreased the binding affinity of ENL-S1 for ENL YEATS. To determine the importance of valine in the truncated peptide context, we synthesized tENL-S1oV1A in which the V1A mutation was introduced into tENL-S1o and assessed its binding to the ENL YEATS domain using AlphaScreen. Intriguingly, the mutation did not affect binding affinity significantly, as the V1A variant displayed comparable potency to tENL-S1o, as shown in Figure S20. This finding indicates that while the valine residue may influence the structure of a full-length 7-mer peptide, it is not critical in the truncated version. Overall, these simulation insights contribute to our understanding of **tENL-S1f**’s high affinity and specificity for the ENL YEATS domain as opposed to the AF9 YEATS domain. Nevertheless, crystallographic studies are required to fully elucidate the binding dynamics.

## Conclusion

Epigenetic proteins including epigenetic writers, erasers, and readers represent a plethora of prospective therapeutic targets. Recently, there has been significant progress in the discovery and clinical implementation of various epigenetic pharmaceuticals, including tazemetostat and virinostat.^51,52^ However, most current epigenetic pharmaceuticals target epigenetic writers and erasers. Epigenetic writers and erasers are enzymes with well-shaped active sites for catalysis, making the development of inhibitors relatively straightforward. However, reader proteins typically interact with chromatin or regulatory proteins through a shallow cavity or a binding groove, which is delineated by an extensive interaction surface area. This expansive and intricate interface complicates the development of small molecule drugs aimed at these proteins.^53^ Adding to the complexity is the presence of multiple homologous reader proteins, which significantly hampers the development of selective ligands targeting a particular reader protein. Although homologous reader proteins possess analogous channels, the structures surrounding these channels engaging with target proteins are diverse. Consequently, a viable strategy for devising selective ligands for a reader protein might entail not only the reader channel but also the surrounding interaction surface area. We suggest that employing a phage display library, incorporating a genetically encoded ncAA interacting specifically with the reader channel, could meet the requirements for generating highly selective ligands for a reader protein.

In the present study, we used the ENL YEATS domain as a representative reader protein to successfully validate our proposed method, which resulted in the identification of highly potent and specific inhibitors. We effectively used BuK as a warhead to guide its containing peptides displayed on phage toward the reader channel of ENL YEATS and, in the process, discovered a peptide inhibitor named **ENL-S1**. The butyryl group in ENL-S1 was then replaced with entities that possess a conjugated system in order to leverage the π–π–π stacking interaction with ENL YEATS. This switch enhanced binding affinity, supporting our theory that BuK occupies the reader channel and serves as a guide for peptides to bind ENL YEATS. Interestingly, due to the selective interactions between the peptide residues and the surrounding area of the reader pocket, **ENL-S1** and its derivatives demonstrated exceptional selectivity for ENL YEATS over AF9 YEATS. Optimization of the acyl-lysine side chain resulted in an inhibitor with an extraordinarily impressive potency and a *K*_D_ value of 2 nM. One interesting feature of ENL-S1 is the role that cysteine plays in binding. Attempts to oxidize both the full length and truncated peptides to a dimer were futile and resulted in precipitation, indicating that the active form in solution is the monomer. Mutation of the cysteine to alanine, however, determined that the cysteine was necessary for potent inhibition of the interaction between ENL and an acylated peptide. Crystallographic studies may give more information on exactly the role that cysteine is playing in the binding. The results from the alanine scan also helped to improve cell permeability through generation of truncated peptide derivatives, which was demonstrated using fluorescent microscopy assays. Currently, we are investigating the mechanism of entry and hope to report upon this in future studies. We assessed the resultant peptides using a newly developed NanoBRET assay for their cellular target engagement, where we identified a low-micromolar ENL inhibitor, **tENL-S1f**. Further analysis confirmed that **tENL-S1f** selectively targets ENL over other YEATS domains and effectively inhibits ENL target gene expression in cells as well as leukemia cell growth.

This study stands as the pioneering effort showcasing the feasibility of using phage display, in tandem with a genetically encoded ncAA acting as a binding anchor, for developing selective inhibitors for an epigenetic reader protein. Over the past years, through expanding the genetic code, our team and others have successfully incorporated a broad range of ncAAs, including acylated and methylated lysines.^13,14,16,18,54^ These ncAAs often act as histone markers and facilitate well-defined interactions with epigenetic reader proteins, such as bromodomains and chromodomains. These interactions can be effectively harnessed in a high-throughput manner through phage display, leading to the identification of potent and selective peptide therapeutics. We anticipate that this ncAA-incorporating phage display approach, due to its wide applicability, will have extensive use in this field.

## Supporting Information Available

Supplementary Methods, Figures S1–S39, and Supplementary Scripts (PDF)
ENLR3 (XLSX)
ENLcontrolR3 (XLSX)
